# Acute Appendicitis as an Incidental Diagnosis of Situs Inversus Totalis: A Case Report and Literature Review

**DOI:** 10.7759/cureus.115963

**Published:** 2026-09-08

**Authors:** Vanessa Galvan, Luis F Ochoa, Katherine S Trejos Guzman, Paola R Zuniga, Dariana Mena, Walter V Jovel Suazo, Claudia Sanchez, Rishita Dave, Gina Ibarra Castro, Yomayra Negron

**Affiliations:** 1 Surgery, Hospital General Regional #1, Instituto Mexicano del Seguro Social (IMSS), Ciudad Obregón, MEX; 2 Surgery, Hospital General Instituto de Seguridad y Servicios Sociales de los Trabajadores del Estado (ISSSTE) Presidente Lázaro Cárdenas, Chihuahua, MEX; 3 Surgery, Universidad Nacional Autónoma de Nicaragua, Managua, NIC; 4 General Practice, Universidad Nacional Autónoma de Honduras, Tegucigalpa, HND; 5 Surgery, Universidad Autónoma de Baja California, Mexicali, MEX; 6 Surgery, Universidad de El Salvador, San Salvador, SLV; 7 Medicine and Surgery, Universidad Catolica Boliviana, Santa Cruz, BOL; 8 Internal Medicine, University of Medicine and Health Sciences, Camps, KNA; 9 Surgery, Universidad Autónoma de Guadalajara, Guadalajara, MEX; 10 Psychiatry, First Hospital Panamericano, San Juan, PRI

**Keywords:** diagnosis of acute appendicitis, left sided abdominal pain, left-sided acute appendicitis, open appendectomy technique, situs-inversus-totalis

## Abstract

Acute appendicitis in patients with situs inversus totalis (SIT) represents a diagnostic challenge because the clinical presentation frequently differs from the classic right lower quadrant pain pattern. We present the case of a 37-year-old male with acute appendicitis associated with previously undiagnosed SIT, identified incidentally during diagnostic imaging. The patient presented with left lower quadrant abdominal pain, leukocytosis, and localized tenderness. Computed tomography revealed dextrocardia and left-sided inflammatory changes compatible with acute appendicitis in the setting of SIT. Laparoscopic appendectomy was successfully performed with modified port placement according to the reversed anatomy, with favorable postoperative recovery.

This case highlights the importance of considering anatomical anomalies in patients presenting with atypical abdominal pain. Imaging studies, particularly computed tomography, play a crucial role in establishing an early diagnosis and preventing complications such as perforation or generalized peritonitis. Furthermore, laparoscopy offers both diagnostic and therapeutic advantages in patients with situs inversus. Awareness of this uncommon entity is essential for surgeons and emergency physicians in order to avoid delays in diagnosis and treatment.

## Introduction

Situs inversus totalis (SIT) is a rare congenital anomaly characterized by a complete mirror-image transposition of thoracic and abdominal organs, with an estimated incidence ranging from one in 10,000 to one in 50,000 individuals [1]. This condition may occur as an isolated anomaly or as part of genetic disorders associated with defects in ciliary motility, such as primary ciliary dyskinesia and Kartagener syndrome [1,2]. Although most individuals with SIT remain asymptomatic and are diagnosed incidentally, the altered anatomical configuration may represent a significant challenge during the evaluation and management of acute abdominal conditions.

Acute appendicitis is one of the most frequent causes of acute abdomen requiring surgical intervention worldwide. However, its occurrence in patients with SIT is uncommon, with only a limited number of cases reported in the literature [3]. The atypical anatomical position of the cecum and appendix, usually located in the left iliac fossa, frequently results in an atypical clinical presentation that may delay diagnosis and increase the risk of complications. Left-sided abdominal pain in these patients can mimic other conditions, including acute diverticulitis, ureteral colic, inflammatory bowel disease, and gynecological disorders, making clinical suspicion essential [4].

The diagnosis of acute appendicitis in patients with SIT requires a high index of suspicion and careful anatomical assessment. Although physical examination findings may be misleading, imaging studies have become fundamental for accurate diagnosis. Abdominal ultrasonography can provide initial evaluation; however, computed tomography (CT) is considered the most reliable imaging modality because it allows simultaneous identification of appendiceal inflammation and confirmation of the underlying anatomical abnormality [5]. Recognition of SIT before surgical intervention facilitates operative planning, reduces intraoperative uncertainty, and may decrease the risk of complications.

The surgical management of acute appendicitis in patients with SIT has evolved with advances in minimally invasive techniques. Laparoscopic appendectomy is currently considered a safe and effective approach because it provides diagnostic confirmation and therapeutic treatment while allowing adequate visualization of the altered abdominal anatomy [6]. Nevertheless, laparoscopic surgery may not be available in all healthcare environments, particularly in institutions with limited resources. In such scenarios, open appendectomy remains a valid treatment option, provided that surgeons are familiar with the mirror-image anatomical orientation.

This case is clinically relevant because acute appendicitis in the setting of SIT poses distinctive diagnostic and surgical challenges, particularly in resource-limited environments. Although individual cases have been previously reported, this uncommon anatomical configuration may lead to diagnostic delay and requires adaptation of conventional surgical approaches. Its documentation contributes to increasing awareness of atypical presentations, reinforces the importance of recognizing anatomical variations before surgery, and illustrates the continued role of open surgery when minimally invasive resources are unavailable.

## Case presentation

In April 2026, a 37-year-old male patient presented to the emergency department of our hospital with sudden-onset abdominal pain localized to the left iliac fossa, rated 8/10 on the Visual Analog Scale (VAS), with six hours of evolution, accompanied by nausea and multiple episodes of vomiting of gastric contents; he denied any other associated symptoms. No significant past medical history was mentioned. On directed physical examination, the abdomen was globose due to adipose panniculus, non-distended; bowel sounds were present but decreased on auscultation, tympanic on percussion, with tenderness to palpation in the left iliac fossa and positive rebound tenderness, without involuntary abdominal guarding or classic appendiceal signs. 

Due to the clinical presentation and the patient’s age, diverticulitis was initially suspected. Medical management consisting of analgesics and antibiotics was initiated. The following blood tests were performed (Table 1); C-reactive protein, erythrocyte sedimentation rate, and procalcitonin were not requested as part of the preoperative protocol. 

**Table 1:** Laboratory tests

Value	Result	Normal Range
Hemoglobin	16.4 g/dL	13.5-17.5
Hematocrit	49.5%	41-53
Leukocyte count	4,950 uL	4,500-11,000
Platelet count	311,000 uL	150,000-450,000
Blood Urea Nitrogen	16.8 mg/dL	8-18
Urea	36 mg/dL	15-43
Creatinine	1 mg/dL	0.6-1.4
Uric Acid	5.4 mg/dL	3.5-8.5
Sodium	138 mmol/L	137-148
Potassium	4.3 mmol/L	3.5-5.1
Calcium	9.5 mmol/L	8.4-10.2
Chloride	102 mmol/L	98-110
Phosphorus	3.5 mmol/L	2.4-4.5

The patient had an Alvarado score of 2/10, based only on the presence of nausea/vomiting and rebound tenderness [7]. Furthermore, the AIR score could not be fully calculated because C-reactive protein and neutrophil percentage were not obtained.

Additionally, an abdominopelvic computed tomography scan was performed, revealing dextrocardia (Figure 1), as well as the spleen and gastric chamber located on the right side and the liver on the left side (Figure 2), leading to the diagnosis of SIT.

**Figure 1:**
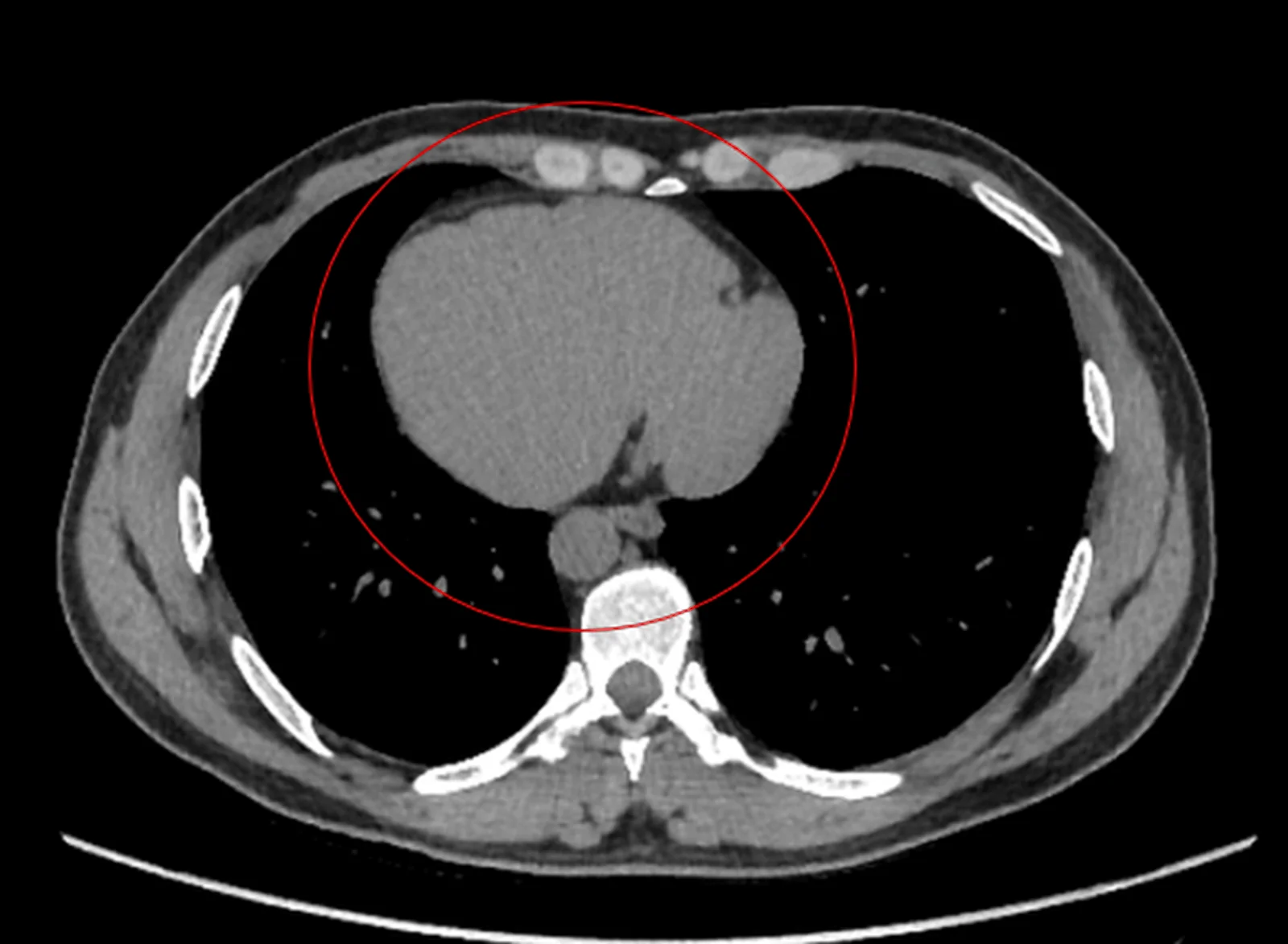
Non-contrast chest computed tomography. Non-contrast chest computed tomography demonstrating dextrocardia (red circle).

**Figure 2:**
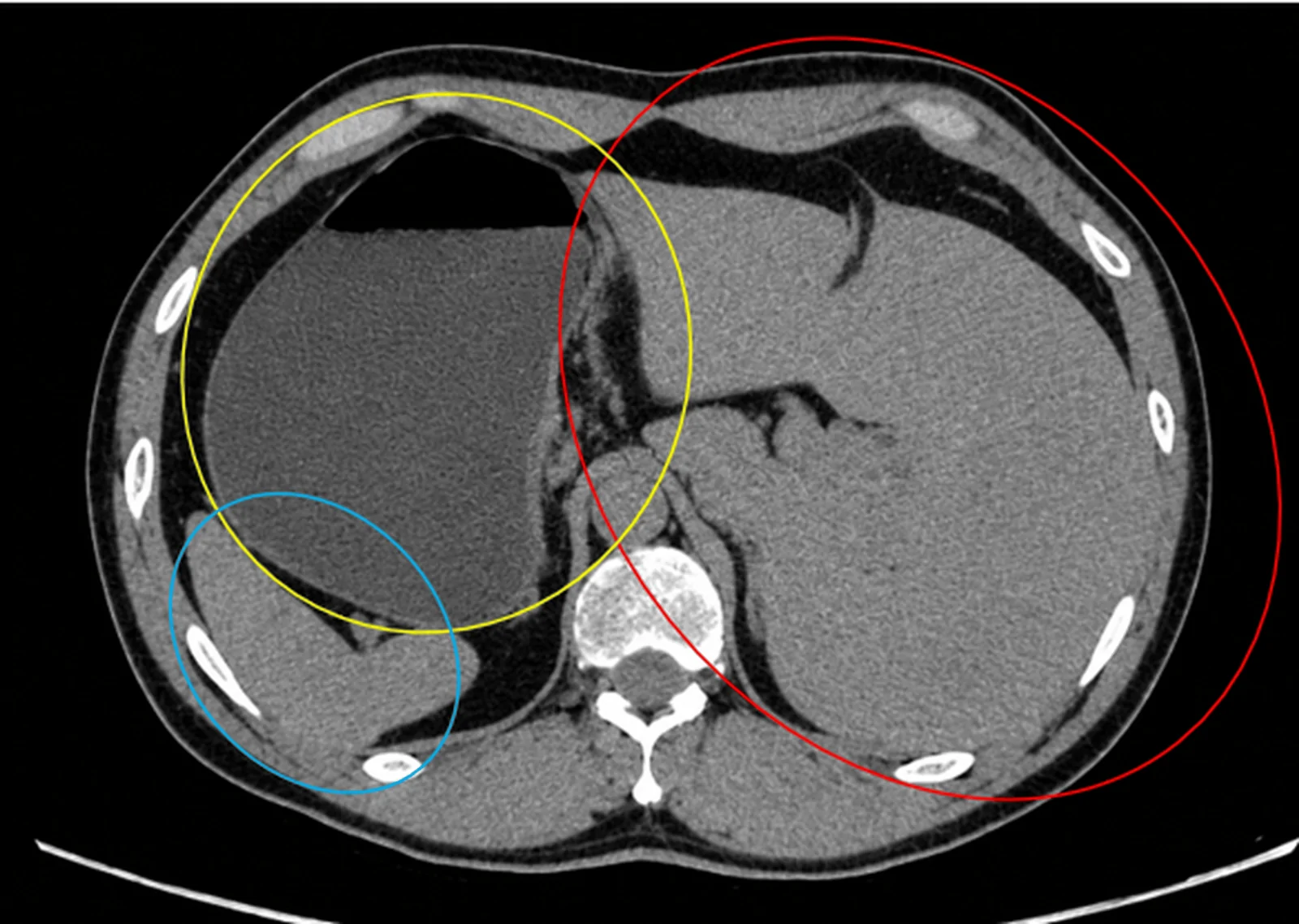
Non-contrast abdominal computed tomography. Non-contrast abdominal computed tomography demonstrating the stomach (yellow circle) and spleen (blue circle) on the right side and the liver (red circle) on the left side.

Furthermore, a left-sided cecal appendix was identified, showing appendiceal wall thickening of 1 cm and periappendiceal fat stranding (Figures 3, 4), findings consistent with acute appendicitis.

**Figure 3:**
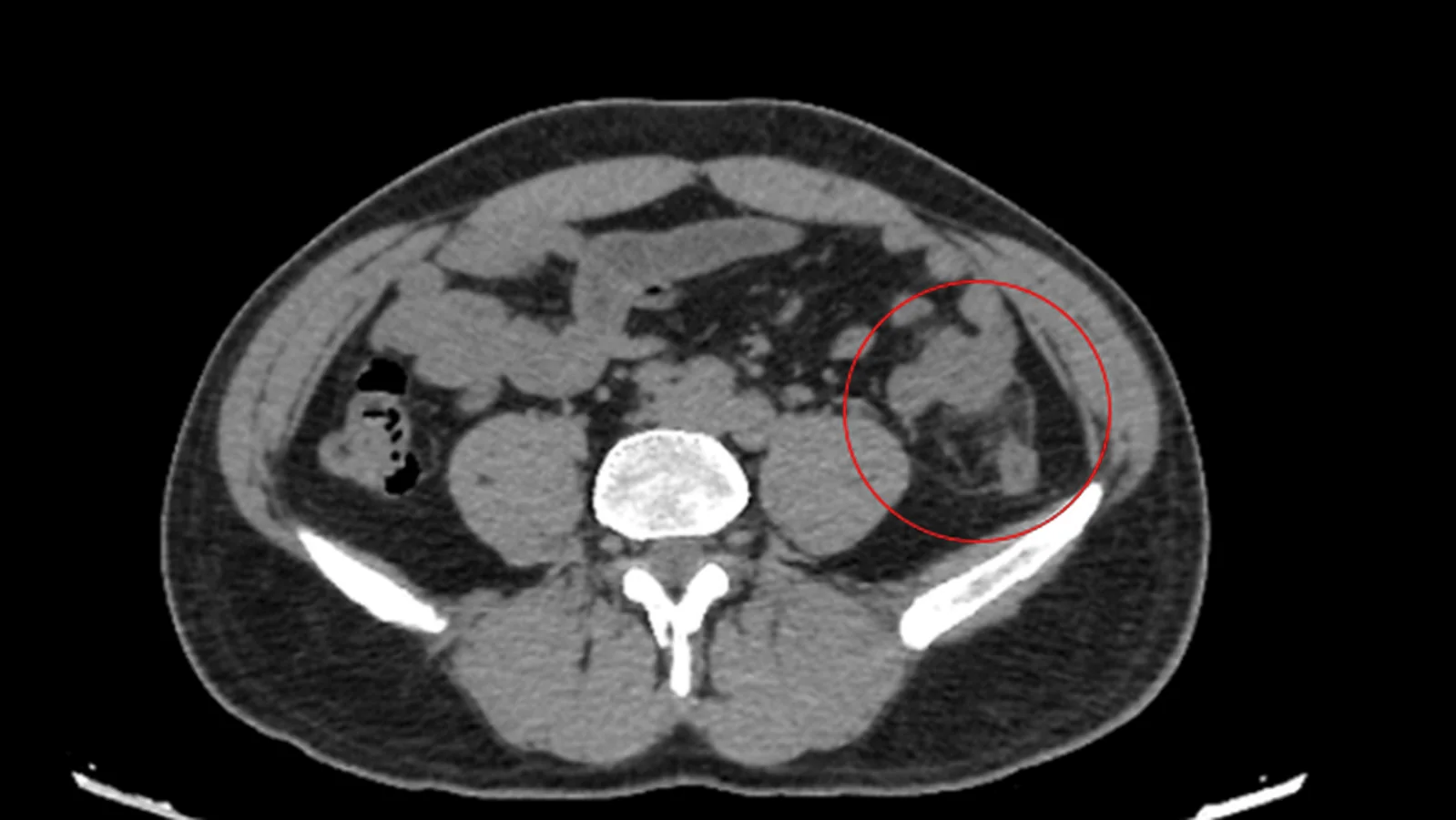
Non-contrast abdominal computed tomography. Periappendiceal fat stranding (red circle) is observed on the left side due to the presence of situs inversus totalis.

**Figure 4:**
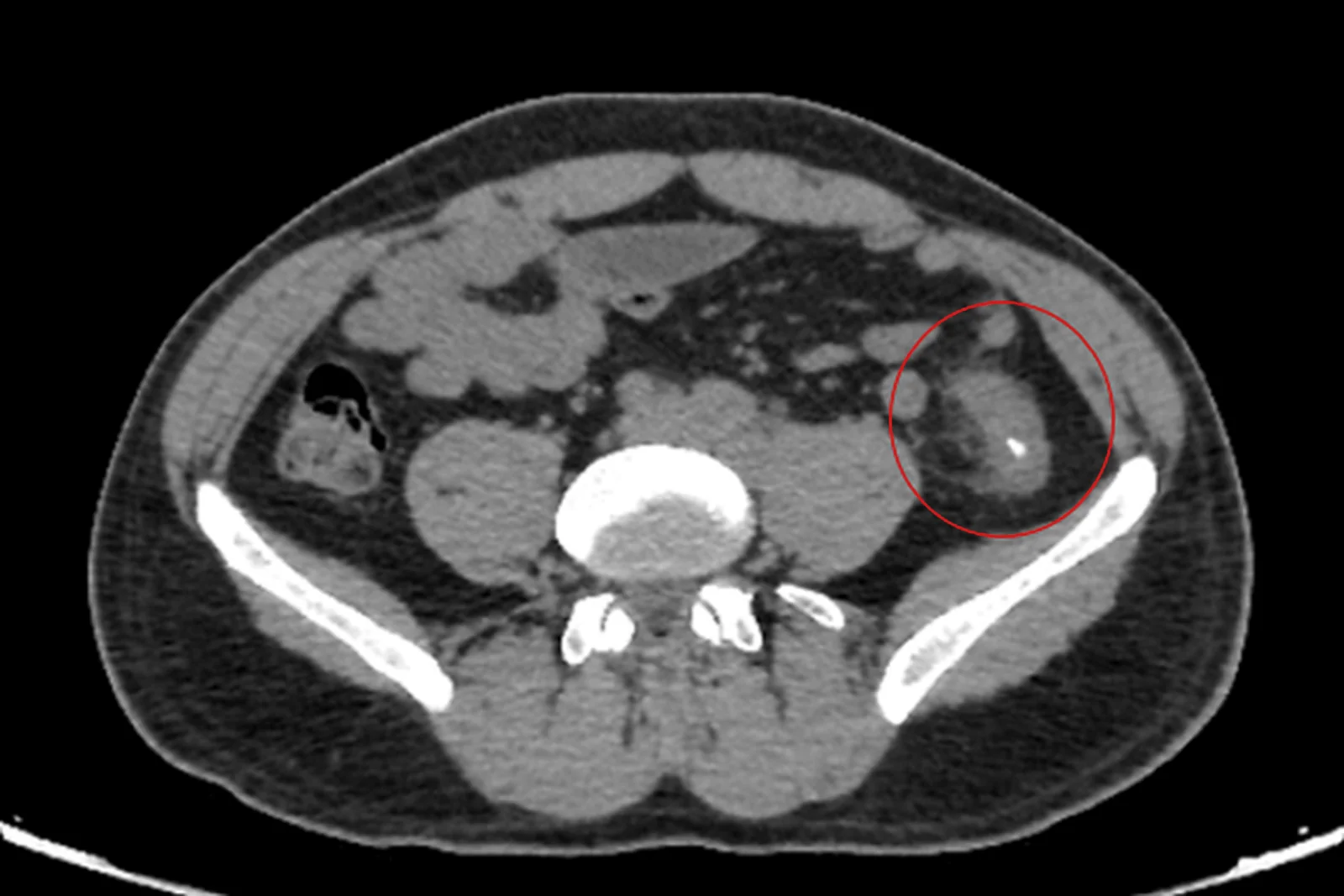
Non-contrast abdominal computed tomography. An appendicolith is identified within the appendix, along with appendiceal wall thickening and periappendiceal fat stranding (red circle).

Due to these findings, an urgent operating room was requested for open appendectomy. A left-sided Rockey-Davis incision was performed due to the anatomical presentation, followed by dissection through the tissue planes until entering the abdominal cavity, where a phase II (edematous) cecal appendix was identified (Figures 5, 6). The mesoappendix was dissected, clamped, ligated, and resected in an antegrade fashion until reaching the appendiceal base. Appendectomy was then performed (Figure 7), and the stump was managed using invagination of the stump with purse-string suture. Hemostasis was verified, and closure was performed with continuous Vicryl 1-0 sutures for the aponeurosis, inverted Vicryl 2-0 sutures for the subcutaneous cellular tissue, and Nylon 3-0 for the skin. Surgical wounds were covered, and the procedure was concluded. Transoperative bleeding was 150 mL. 

**Figure 5:**
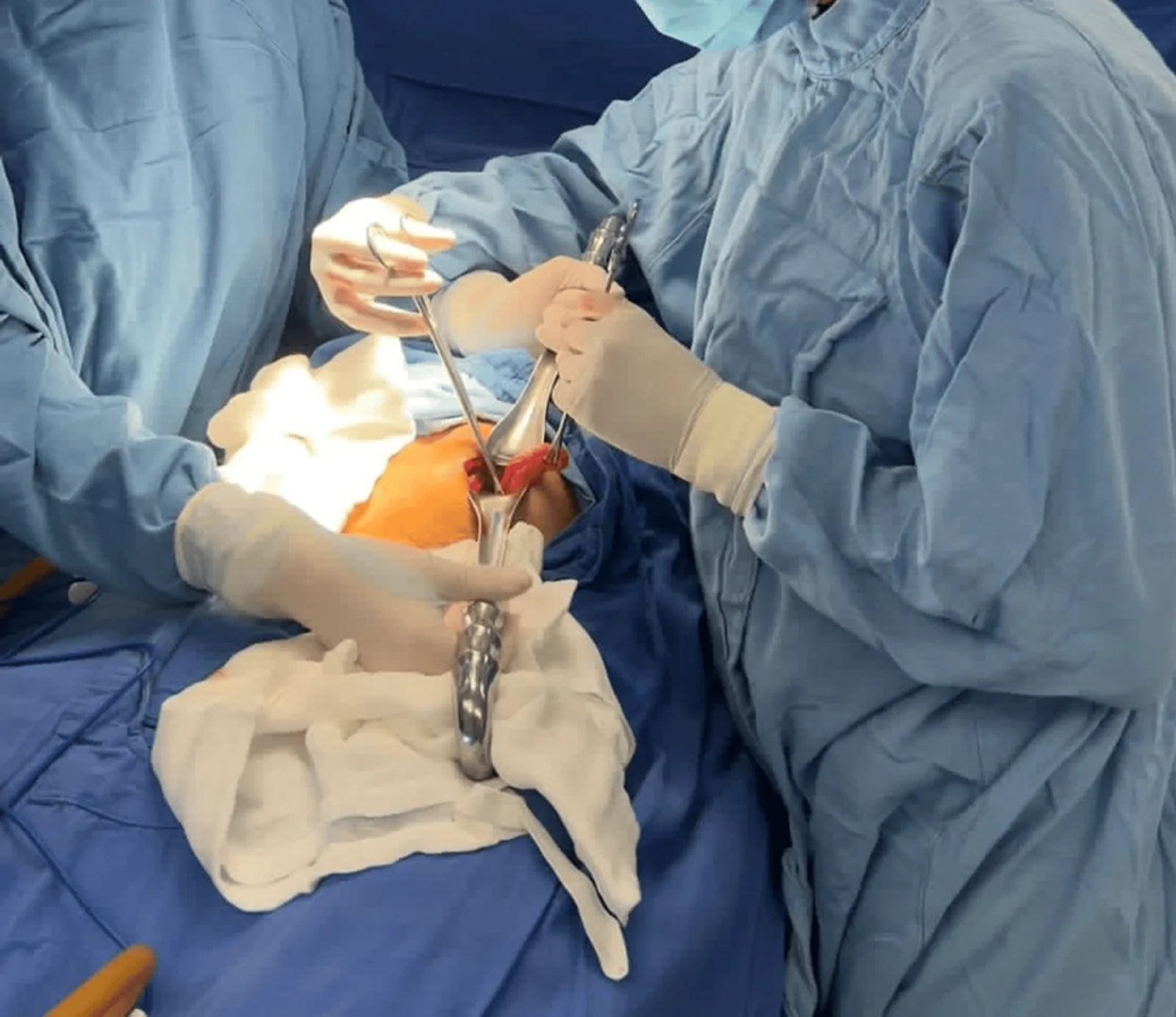
Left side open appendectomy. Intraoperative image demonstrating a left-sided open appendectomy performed due to situs inversus totalis.

**Figure 6:**
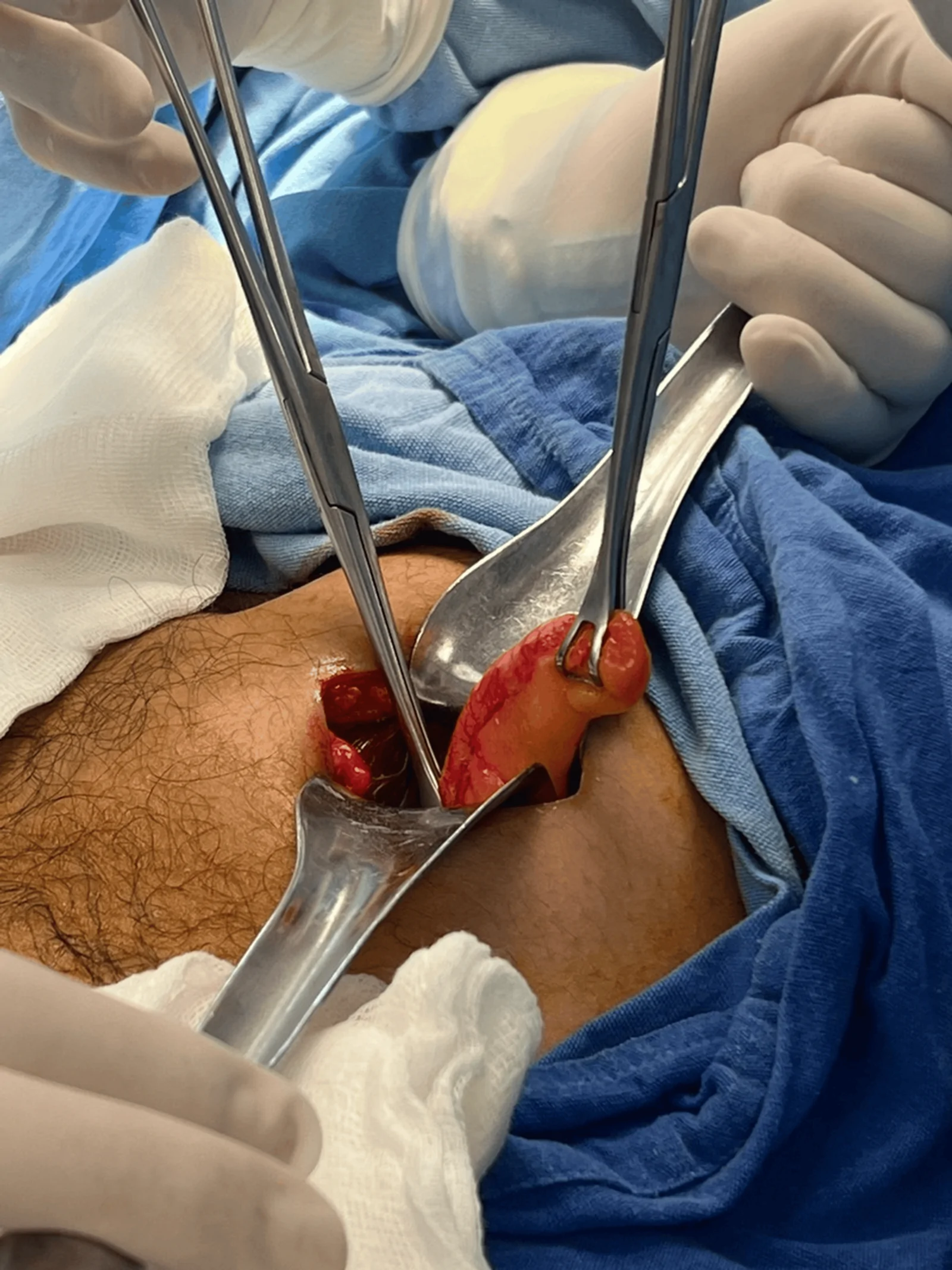
Left side open appendectomy. Intraoperative image demonstrating a left-sided open appendectomy using Rocky Davis technique, performed due to situs inversus totalis.

**Figure 7:**
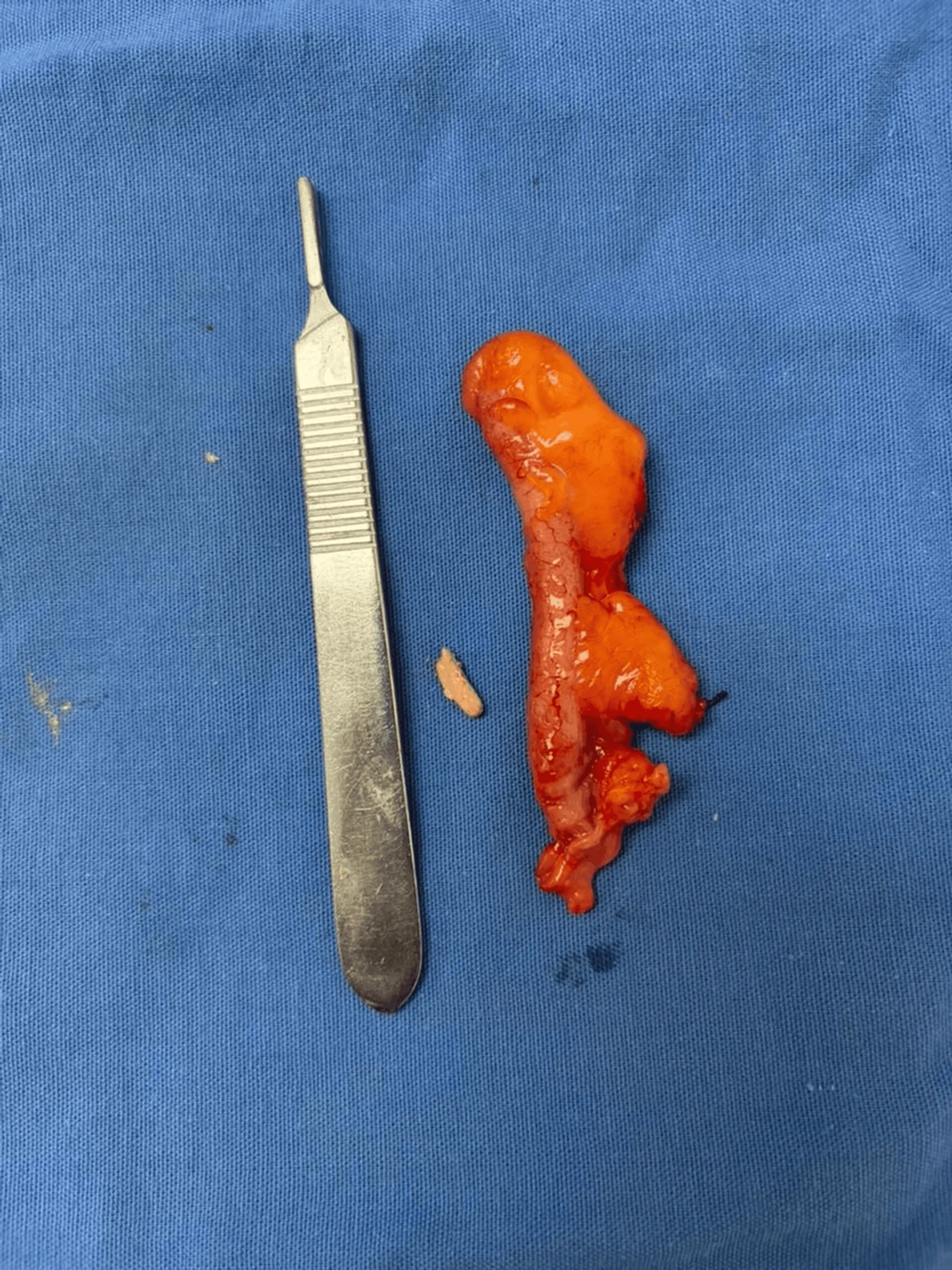
Macroscopic image of the appendix following open appendectomy. Acute phase II appendicitis is observed, characterized by marked inflammatory changes, including edema and erythema of the appendix.

The patient had no intraoperative complications and experienced a favorable postoperative course characterized by tolerance to oral intake, bowel movements, and passage of flatus, which allowed discharge one day after the surgical procedure.

Two months after the procedure, the histopathological examination results were obtained, as follows. For the macroscopic description, a cecal appendix measuring 5.5 × 1.2 cm is received. It has a grayish surface covered with fibrinopurulent plaques. On sectioning, the wall measures 0.2 cm and is light brown and soft; the lumen is dilated by fecal material. The appendectomy specimen result was acute appendicitis and periappendicitis. 

The patient returned for follow-up one month after surgery. He reported being asymptomatic and had experienced a favorable postoperative course. Physical examination revealed a well-healed surgical wound. Additionally, the histopathological examination revealed benign findings. Based on the clinical history, patient interview, physical examination, and histopathological findings, he was discharged from our service.

## Discussion

The diagnosis of acute appendicitis in patients with SIT represents a significant clinical challenge because the classic presentation of right lower quadrant pain is frequently absent. Instead, inflammation of the appendix located in the left iliac fossa commonly produces left-sided abdominal pain, which may resemble other conditions such as acute diverticulitis, renal colic, inflammatory bowel disease, or gynecological pathology [4]. Nevertheless, atypical presentations have also been reported, including patients with right-sided abdominal pain despite complete visceral transposition. This phenomenon may be explained by variations in visceral innervation, individual anatomical differences, or atypical appendix position [5].

Akbulut et al. performed one of the largest reviews of left-sided appendicitis associated with SIT, analyzing 63 published cases and emphasizing the diagnostic difficulties caused by atypical symptom localization [6]. More recent systematic reviews have demonstrated that most patients are diagnosed only after presenting with abdominal symptoms, with a significant proportion unaware of their anatomical abnormality before the acute episode [8]. 

Acute appendicitis with normal WBC occurs in 10-20% of cases, especially early in the disease course. Normal inflammatory markers do not exclude the diagnosis of acute appendicitis. Although leukocytosis and elevated C-reactive protein levels are frequently observed, their diagnostic accuracy is insufficient when interpreted in isolation, and a subset of patients with histologically confirmed acute appendicitis may present with a normal white blood cell count [9]. Previous studies have specifically documented acute appendicitis in patients with normal leukocyte counts, highlighting that the absence of leukocytosis should not be used as an isolated criterion to rule out the disease [8]. Furthermore, inflammatory biomarkers have variable diagnostic performance and should therefore be interpreted in conjunction with the patient's clinical presentation and other diagnostic findings [10]. Current evidence-based guidelines emphasize a multimodal diagnostic approach incorporating clinical assessment, risk stratification, and imaging to improve diagnostic accuracy [11]. When strictly applying the conventional Alvarado score, the patient had a score of 2/10, based only on the presence of nausea/vomiting and rebound tenderness [12]. However, this low score should be interpreted cautiously because two major components of the Alvarado score - migration of pain to the right lower quadrant and right lower quadrant tenderness - are anatomically oriented toward the usual position of the appendix [12,13]. In patients with SIT, left-sided appendiceal location may therefore reduce the applicability of conventional clinical scoring systems if interpreted literally. Furthermore, the AIR score could not be fully calculated because C-reactive protein and neutrophil percentage were not obtained [13]. These limitations emphasize the importance of integrating clinical assessment with imaging findings in patients with atypical anatomy rather than relying exclusively on conventional appendicitis scoring systems [12,13]. In patients with atypical anatomical conditions such as SIT, this integrated approach becomes particularly relevant because the unusual location of symptoms may further complicate the clinical recognition of acute appendicitis.

Although contrast-enhanced computed tomography would generally be preferred in the evaluation of left lower quadrant abdominal pain and suspected diverticulitis, intravenous contrast was not administered in this case because of limitations in its availability at our resource-limited institution. Contrast media are not consistently available, particularly during certain shifts, and therefore a non-contrast abdominopelvic CT scan was performed as the most readily available cross-sectional imaging modality at the time of presentation. Ultrasonography was also considered; however, its diagnostic performance is highly operator-dependent. At our institution, radiologists and personnel with dedicated expertise in abdominal ultrasonography are not consistently available during all shifts. Given these resource constraints and the initial clinical suspicion of diverticulitis in an adult patient presenting with left lower quadrant pain, non-contrast CT was considered the most feasible imaging modality available at that time. These institutional limitations should be acknowledged when interpreting the diagnostic approach used in this case.

CT has become the preferred imaging modality because it provides detailed visualization of both the inflammatory process and the anatomical configuration of the abdominal organs [14]. In this case, non-contrast CT scan was sufficient to identify the abnormal left-sided anatomy and inflammatory changes that ultimately led to the diagnosis of acute appendicitis in the setting of SIT.

The surgical management of appendicitis in SIT has evolved considerably with the development of minimally invasive techniques. Laparoscopic appendectomy is currently considered a safe and effective approach, providing diagnostic confirmation, adequate visualization of the abdominal cavity, and therapeutic treatment through minimally invasive access [15]. Several authors have reported successful laparoscopic appendectomy in patients with SIT; however, the procedure requires adaptation to the reversed anatomy. Surgeons must consider the location of the trocars, the orientation of the operating surgeon, and the possibility of altered vascular or mesenteric anatomy [16].

Despite the advantages of laparoscopy, access to minimally invasive surgery is not universal. Limitations in equipment availability, institutional resources, or surgical expertise may require alternative approaches [16]. In the present case, laparoscopic surgery was not feasible because of the absence of appropriate equipment; therefore, an open appendectomy was performed through a modified left lower quadrant incision based on the patient's anatomical configuration. This approach allowed adequate exposure, identification of the appendix, and safe completion of the procedure without complications.

Previous reports have demonstrated that open appendectomy remains a reliable option in patients with SIT, particularly when performed by surgeons familiar with the altered anatomical orientation; although the lack of laparoscopic visualization may theoretically increase intraoperative difficulty, careful preoperative imaging evaluation and anatomical awareness allow successful surgical management [17,18]. Table 2 compares the present case with selected reports from the recent literature. In resource-limited environments, open appendectomy remains a viable and safe alternative, provided surgeons are aware of the reversed anatomy. 

**Table 2:** Surgical management comparison with the previous literature.

Study	Age/Sex	Clinical presentation	Imaging	Surgical management	Outcome
Pipal et al. [19]	36-year-old male.	Left lower abdominal pain for 2 days; left iliac fossa tenderness.	Ultrasonography followed by contrast-enhanced CT confirming situs inversus totalis and acute appendicitis.	Laparoscopic appendectomy.	Uneventful recovery.
Abdulla et al. [20]	65-year-old male.	Left lower quadrant abdominal pain.	CT demonstrated a swollen left-sided appendix with periappendiceal fat stranding and situs inversus totalis.	Laparoscopic appendectomy.	Uneventful postoperative course; discharged on postoperative day 2.
Mahat et al. [21]	50-year-old male.	Left lower abdominal pain for 7 days associated with fever, nausea, vomiting, and loose stools.	CT confirmed left-sided acute appendicitis in the setting of situs inversus totalis.	Emergency laparoscopic appendectomy.	Favorable postoperative evolution.
Mvoula et al. [22]	45-year-old male.	Severe left lower quadrant abdominal pain, localized tenderness, nausea, and vomiting.	Abdominopelvic CT demonstrated previously undiagnosed situs inversus totalis and acute appendicitis.	Emergency laparoscopic appendectomy.	Uneventful recovery; discharged on postoperative day 2.
Present case	37-year-old male.	Sudden-onset left iliac fossa pain, nausea, multiple episodes of vomiting, rebound tenderness, and normal leukocyte count; diverticulitis initially suspected.	Non-contrast abdominopelvic CT demonstrated situs inversus totalis and a left-sided inflamed appendix with approximately 1-cm wall thickening and periappendiceal fat stranding.	Open appendectomy through a modified left-sided incision because laparoscopic equipment was unavailable.	Favorable postoperative clinical course.

An additional challenge in patients with SIT is the possibility of unexpected intraoperative findings. Variations in vascular anatomy, intestinal fixation, and mesenteric relationships may occur and should be anticipated before surgery. Although these variations are uncommon, awareness of possible anatomical differences reduces operative time and prevents iatrogenic complications [22].

This case also emphasizes the importance of considering SIT in the evaluation of patients presenting with atypical abdominal pain. Failure to recognize the condition may result in delayed diagnosis, progression to perforation, abscess formation, or generalized peritonitis. Early imaging confirmation and appropriate surgical planning remain essential factors associated with favorable outcomes.

## Conclusions

Acute appendicitis associated with SIT is a rare entity that poses a significant diagnostic challenge because of its atypical clinical presentation. Left lower quadrant abdominal pain should raise suspicion for possible anatomical variations, particularly when supported by imaging findings suggestive of transposed viscera.

Computed tomography remains the most valuable diagnostic modality for confirming both appendicitis and situs inversus totalis. Although laparoscopic appendectomy is generally considered the treatment of choice, open appendectomy continues to be a safe and effective alternative when laparoscopic equipment or expertise is unavailable. Awareness of this uncommon condition among surgeons and emergency physicians is essential to ensure prompt diagnosis and adequate surgical management.
